# Fulminant Herpes Simplex Virus Type I Encephalitis Despite Maximal Medical Therapy

**DOI:** 10.7759/cureus.2467

**Published:** 2018-04-12

**Authors:** Nakul Katyal, Ather M Taqui, Deborah Tepper, Jonathan M Beary, Christopher R Newey

**Affiliations:** 1 Department of Neurology, University of Missouri, Columbia, USA; 2 Neurology, Cleveland Clinic Ohio; 3 Neurobehavioral Sciences, A. T. Still University

**Keywords:** herpes simplex encephalitis, fulminant herpes encephalitis

## Abstract

Herpes simplex-1 virus encephalitis (HSE) is the most commonly recognized cause of sporadic encephalitis in the United States. Historically HSE has been considered extremely detrimental given the associated relentless neurological deterioration secondary to cerebral edema and status epilepticus. With recent advances in antiviral therapeutics in past decades, the majority of complications can be managed effectively although the associated morbidity and mortality still remains high. The key modifiable factor determining recovery is the rapid initiation of antiviral therapy. We discuss the case of a 19-year-old female with HSE who received standard acyclovir therapy. Despite using recommended dosage and duration of acyclovir, her clinical condition worsened significantly and subsequently required multiple antiviral therapeutics and steroid therapy.

## Introduction

Herpes simplex virus (HSV) infection is the leading cause of necrotizing viral encephalitis in developed countries [1,2]. The incidence ranges from one to two cases per 500,000 population per year [2]. About 90% of herpes simplex virus encephalitis (HSE) cases in adults and children are related to type-1 HSV [3]. HSE is believed to result from reactivation of latent HSV-1 in dorsal root ganglia that subsequently spreads to the central nervous system (CNS) predominantly affecting temporal lobes [2,3]. Primary HSV pharyngitis in adolescent patients can also lead to HSE via cranial nerves 1 or 5. Prompt recognition and treatment is paramount. If HSE is left untreated, the mortality rate may exceed over 70% with less than 3% of such patients returning to baseline function [4,5]. With advances in antiviral therapy neurological complications can be effectively managed. Early intravenous (IV) acyclovir therapy (10 mg/kg/dose every eight hours x 14–21 days) can significantly reduce mortality to 20–30% [6,7]. We describe the case of an immunocompetent female who presented with complaints of headaches, altered mental status, and short-term memory loss and was diagnosed as having severe HSE. Her clinical condition deteriorated despite early acyclovir therapy thus, requiring deviation from standard antiviral therapy.

## Case presentation

**Figure 1:**
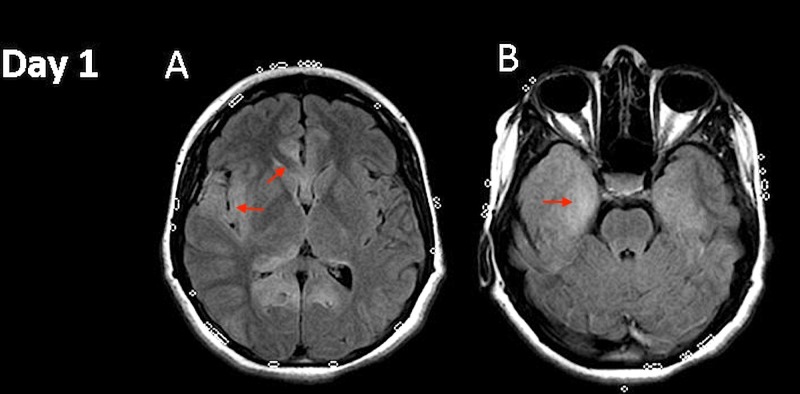
Magnetic resonance imaging (MRI). Hospital day 1 fluid attenuated inversion recovery (FLAIR) changes are noted in the mesial frontal and temporal regions and right insula (A and B).

A 19-year-old female college student presented to the emergency department (ED) with complaints of headaches, altered mental status, and short-term memory loss of four days duration. She had a known history of hypothyroidism and had been non-compliant with her thyroid medication for over one year. Her thyroid stimulating hormone (TSH) level on admission was 220 U/ml (Normal range: 0.3–5 U/ml). Her vital signs were within normal limits. On examination, she had moderate myxedematous features, hoarse voice, and was able to follow only simple commands. Behavioral observations were significant for hyperorality and hypersexuality suggesting Kluver-Bucy syndrome. Lumbar puncture (LP) on day 1 of hospitalization (~5 days from symptom onset) showed 820 white blood cells, 63 red blood cells (RBC), elevated protein of 73 mg/dl, and glucose of 50 mg/dl. Meningoencephalitis was diagnosed and she was started on IV acyclovir at 10 mg/kg/dose every eight hours along with vancomycin and ceftazidime. Magnetic resonance imaging (MRI) of the brain showed fluid-attenuated inversion recovery (FLAIR) sequence hyperintensities in bilateral mesial temporal lobes extending to frontal lobes (Figure 1A, 1B).

**Figure 2:**
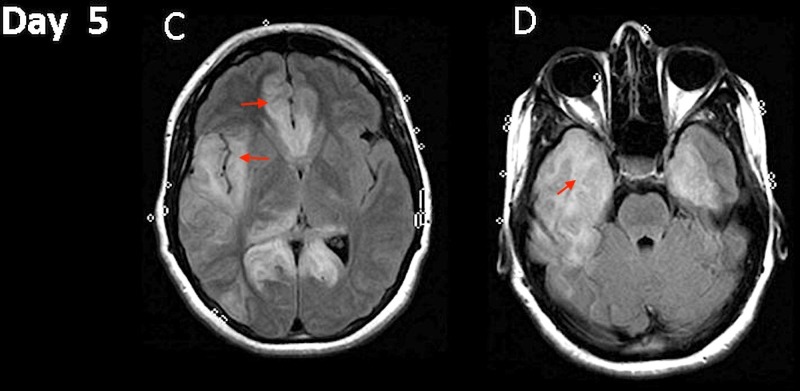
Magnetic resonance imaging (MRI). Hospital day 5 fluid attenuated inversion recovery (FLAIR) changes evolution (C and D).

Microbial cerebrospinal fluid (CSF) analysis revealed a positive HSV-1 polymerase chain reaction (PCR) confirming the diagnosis of HSE. Antibiotics were discontinued and IV acyclovir was maintained at the original dose. Over the next three days, her clinical condition deteriorated with worsening of mental status. On day 5 of admission, repeat MRI showed increased FLAIR changes with extensive involvement of bilateral frontal and bilateral temporal lobes, predominantly right-sided along with right-sided occipital involvement (Figure 2C, 2D).

**Figure 3:**
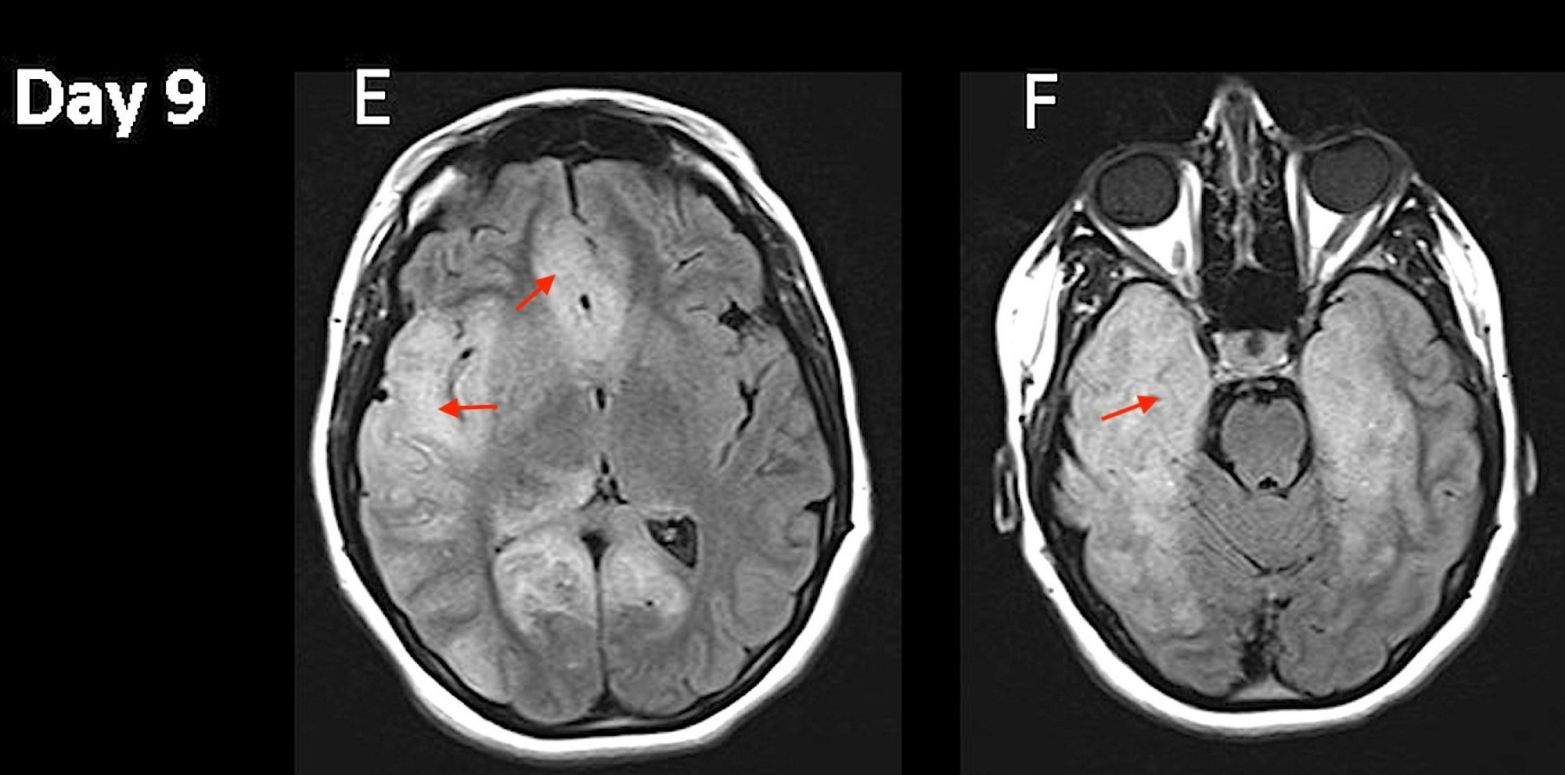
Magnetic resonance imaging (MRI). Hospital day 9 fluid attenuated inversion recovery (FLAIR) changes evolution (E and F).

The IV acyclovir dose was increased to 15 mg/kg/dose every eight hours. On hospital day 9, her mental status had not improved. Repeat MRI showed an increase in FLAIR hyperintensities changes with severe compression of the right lateral ventricle with a mild subfalcine shift to the right, but without distinct transtentorial herniation (Figure 3E, 3F).

**Figure 4:**
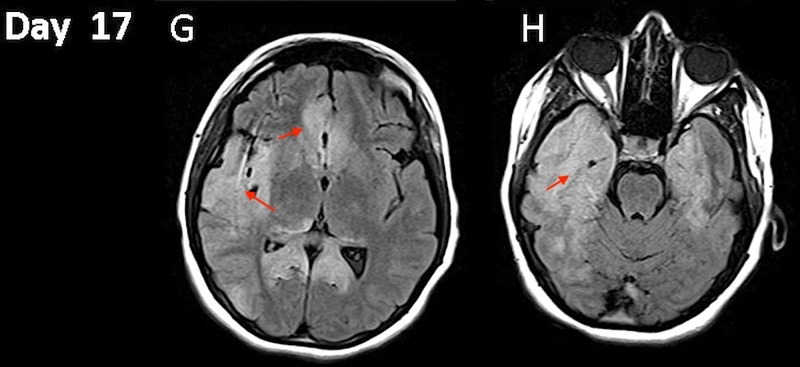
Magnetic resonance imaging (MRI). Hospital day 17 fluid attenuated inversion recovery (FLAIR) changes evolution (G and H).

IV methylprednisolone (1 g daily x five days) was started. Repeat LP on hospital day 11 showed pleocytosis of 608 with RBC count of 15, elevated protein of 114 mg/dl and glucose of 60 mg/dl. The HSV-1 PCR remained positive. On hospital day 12, she had further deterioration of mental status and IV foscarnet 40 mg/kg/dose every eight hours was added to the IV acyclovir. LP and PCR CSF on hospital day 16 showed undetectable HSV-1. A repeat MRI on hospital day 17 showed decreased cerebral edema and mass effect (Figure 4G, 4H).

Although the patient’s mental status improved, she developed severe genital labial swelling as an adverse effect of the foscarnet. She remained on acyclovir at 10 mg/kg/dose every eight hours for a total of 30 days. At the time of discharge after 17 days of hospitalization (~22 days from symptom onset), neurological deficits were short-term memory loss, hyperorality, and irritability.

## Discussion

This case highlights an aggressive form of HSE in a 19-year-old female with clinical and neuroradiological decline despite early acyclovir therapy. Although she subsequently received unconventional therapeutic course of high dose IV acyclovir with addition of foscarnet and methylprednisolone, the patient still experienced disabling residual neurological deficits.

Majority of the literature on standard treatment of HSE is derived from two randomized controlled trials published in the 1980s. One was conducted by Whitley et al. which enrolled 69 brain biopsy-proven HSE patients and compared vidarabine with acyclovir therapy. A significantly lower six-month mortality (54% vs 28%, respectively) and morbidity was seen with acyclovir therapy [7]. Similarly, Skoldenberg et al. conducted a similar study comparing vidarabine and acyclovir and found significantly lower six-month mortality (54% vs 19%, respectively) and morbidity with acyclovir therapy [6]. Both trials used an IV acyclovir dosage of 10 mg/kg/dose every eight hours for a duration of 10 days. Acyclovir dramatically improved mortality rate of untreated HSE patients from 70% to 20–30% [6,7]. Acyclovir selectively inhibits the HSV specific DNA polymerase, thus halting viral replication. It is activated exclusively in HSV-infected cells which makes it relatively non-toxic to normal cells lacking the HSV thymidine kinase enzyme [8].

Ito et al. reported six patients with HSE who suffered from relapse as early as two days after completion of a 10-day course of acyclovir. They found IV acyclovir dosage during initial therapy was significantly lower in the relapse group when compared to the non-relapse group. After administering a repeat course of IV acyclovir at a high dose, 84% of patients showed clinical improvement [9]. Given the clinical and neuroradiological deterioration in our patient on day 9, a decision was made to increase acyclovir dose to 15 mg/kg/dose every eight hours. However, the clinical condition and neuroradiological findings worsened over subsequent days, indicating possible acyclovir resistance. Acyclovir resistance is known to occur in immunocompromised patients who suffer from recurrent herpetic lesions and have received prior repeated acyclovir treatment. Our patient was immunocompetent. However, the occurrence of acyclovir resistance in immunocompetent patients is relatively rare [10]. A large population-based study in the United States reported that 0.2% of immunocompetent hosts harbor HSV strains with borderline resistance [11]. Only one reported case of primary acyclovir resistance in an acyclovir naive immunocompetent patient is known where the patient showed dramatic clinical improvement with the addition of foscarnet in dose of 40 mg/kg every eight hours to the therapeutic regimen. Schulte et al. confirmed resistance via genotypic testing by polymerase chain reaction amplification and sequencing of the thymidine kinase gene of HSV-1 [12]. Our patient was acyclovir naive and immunocompetent. The testing for acyclovir resistance was negative. Most likely our patient was not acyclovir resistant as she only received five days of IV foscarnet. She improved both clinically and radiologically on day 17, when IV foscarnet was stopped.

Steroid therapy has been shown to be an effective adjunctive treatment to antiviral treatment in multiple studies [13,14]. A retrospective study of 45 HSE patients receiving IV acyclovir reported that withholding corticosteroid therapy was an independent predictor of poor outcome [15]. The multicenter, randomized, double-blind, placebo-controlled GACHE trial is a study evaluating the effect of adjunct corticosteroid therapy on morbidity at six months in patients receiving acyclovir [16]. Studies have shown that relapses are more frequent in patients who receive 10 days of acyclovir as compared to patients with longer treatment duration making longer treatment durations of 14–21 days preferable especially in immunocompromised patients [4,9,17]. Our patient received acyclovir treatment for a total of five weeks. This case highlights a fulminant presentation of HSE requiring aggressive unconventional long-term antiviral therapy and now adds to a limited number of cases in the literature describing the management of this rare but potentially life-threatening condition.

## Conclusions

The mortality and morbidity from HSE is significantly high despite the availability of novel antiviral therapeutics. HSE patients with clinical deterioration and worsening MRI findings must be treated aggressively. An increase in both the therapeutic dose and duration of IV antiviral treatment should be considered in resistant cases along with adjunctive corticosteroids.
